# Posterior Reversible Encephalopathy Syndrome (PRES) in Systemic Lupus Erythematosus: A Rare Presentation

**DOI:** 10.7759/cureus.31376

**Published:** 2022-11-11

**Authors:** Sourav Sudan, Navjot Kaur, Saagar Anand, Ashutosh Upadhyaya, Rishabh Taneja

**Affiliations:** 1 Department of Medicine, Government Medical College, Rajouri, IND; 2 Emergency Department, Sadbhavna Medical and Heart Institute, Patiala, IND; 3 Department of Medicine, Government Medical College, Jammu, IND; 4 General Physician, Evergreen Hospital Pvt. Ltd, Parasi, NPL; 5 Intern, Government Multi Specialty Hospital, Chandigarh, IND; 6 Graduate Medical Education, Adesh Institute of Medical Sciences and Research, Punjab, IND

**Keywords:** pres in sle, sle and pres, neuropsychiatric sle, radiological findings in pres, posterior reversible encephalopathy syndrome (pres)

## Abstract

Posterior reversible encephalopathy syndrome (PRES) is a clinical condition that is characterized by intense headache and neurological deficits such as vision loss which are attributed to the vasogenic edema that occurs in the posterior cerebral cortex involving the occipital and parietal lobes. Although the classical demographies that are affected by the PRES are middle-aged postpartum females and those with renal dysfunction, rarely it is also seen in patients with collagen vascular disorders such as systemic lupus erythematosus (SLE). We report a case of PRES in a 32-year-old SLE patient.

## Introduction

Posterior reversible encephalopathy syndrome (PRES) was first described by Hinchey et al. in 1996 as a clinical condition with different presentations but a common radiological characteristic finding of vasogenic edema in the posterior cerebral cortex [1]. 

Although it's not always true, in most cases, PRES is associated with hypertensive emergencies. The various clinical scenarios in which PRES can be found are hypertension (primary or secondary), renal diseases, preeclampsia-eclampsia syndrome and some collagen vascular disorders [2]. The pathophysiology of PRES is linked to hypertension causing increased cerebral blood flow which eventually disrupts the blood brain barrier causing vasogenic edema in the cortex [3,4]. We report a case of PRES in a 32-year-old systemic lupus erythematosus (SLE) patient having both clinical and radiological evidence of PRES.

## Case presentation

**Figure 1:**
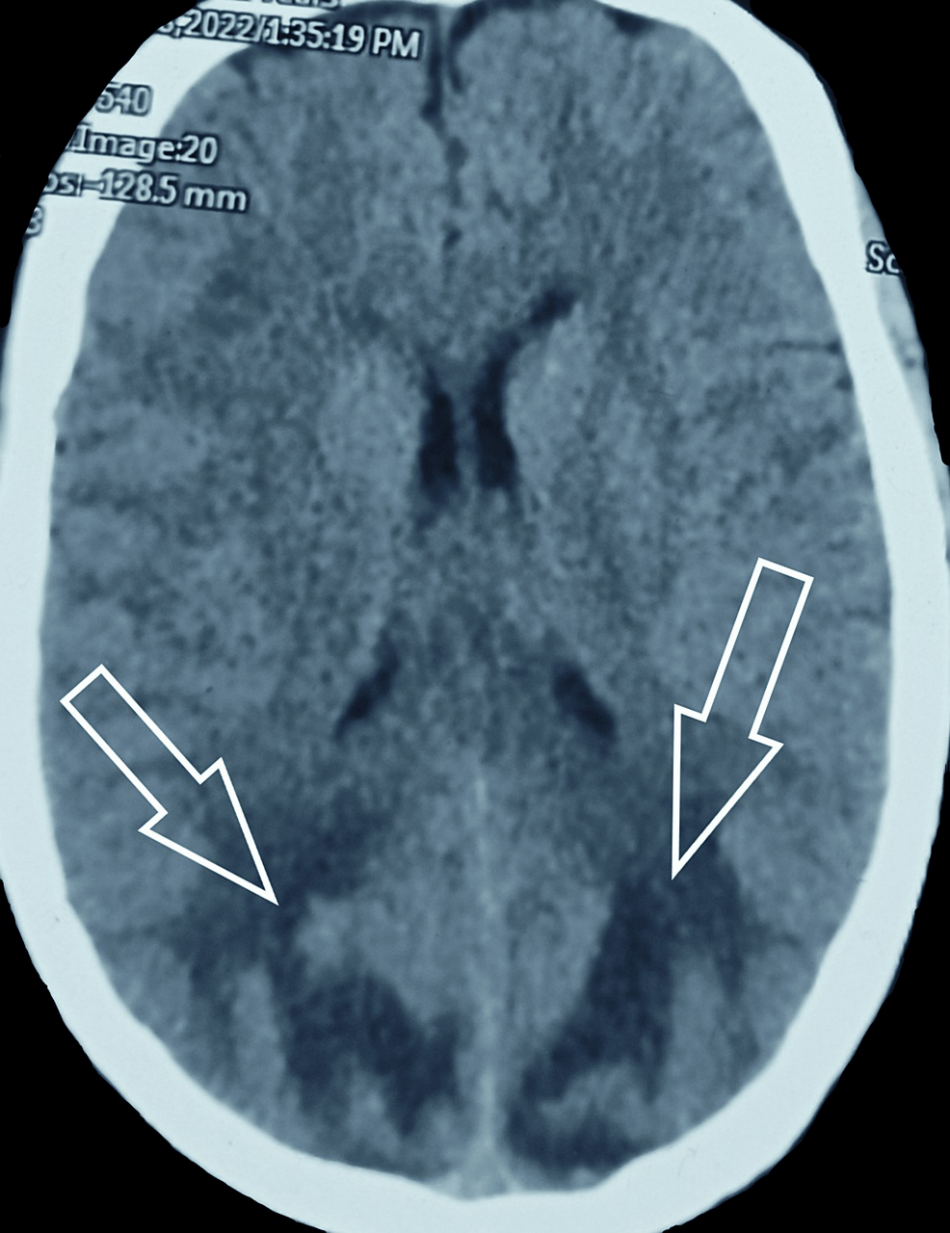
Non-contrast computerized tomography (NCCT) Brain showing symmetrical areas of hypoattenuation in the subcortical white matter of bilateral occipital lobes (white arrows). Grey matter is normal and grey matter-white matter differentiation is maintained. Findings are suggestive of vasogenic edema.

**Figure 2:**
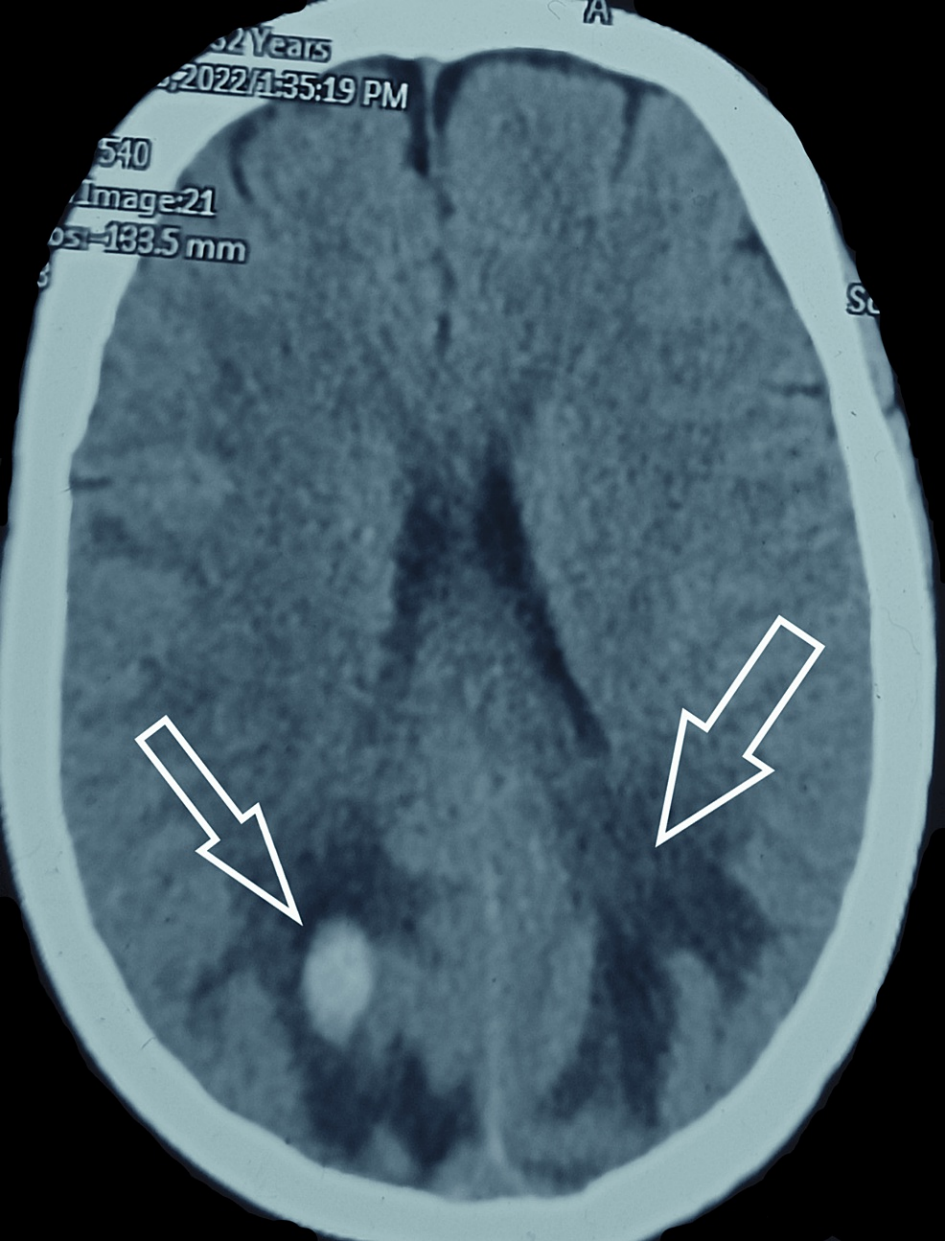
Non-contrast computerized tomography (NCCT) Brain showing symmetrical areas of hypoattenuation in bilateral occipital lobes along with a well-defined hyperdense area in the right side suggestive of hematoma (white arrow on right side) with vasogenic edema (white arrow on left side).

A 32-year-old known case of SLE presented to us with acute loss of vision in both eyes. According to the patient, she was in her usual health three hours prior when she started having an intense headache which was diffuse and associated with it was the blurring of vision which progressed to acute vision loss. On physical examination, she was found to have a blood pressure of 220/120 mm Hg, pulse rate of 80 bpm, and saturation of 97%. Apart from this, she had an erythematous malar rash sparing the nasolabial folds and had onycholysis consistent with her diagnosis of SLE. After initial stabilization with antihypertensives (furosemide 40 mg IV), a noncontrast CT head (NCCT) was done which showed bilaterally symmetrical areas of hypoattenuation in the subcortical white matter of the bilateral occipital lobes (Figure 1). Along with these, a well-defined hyperdense area was also seen in the hypodense area on the right occipital lobe (Figure 2). 

Over the course of her stay in hospital, she was treated with antihypertensives which were switched from injectables to oral in the subsequent days. Alongside, she was given mannitol 10mg three times a day for a day. After 16 hours from admission, her vision started improving with complete recovery over the next eight hours. Correlating the reversible vision loss with the findings of vasogenic edema on NCCT, the diagnosis of PRES was made. The patient was monitored for two more days and was subsequently discharged. She is currently stable and there have been no such episodes since her discharge from the hospital.

## Discussion

PRES syndrome usually affects individuals who have underlying comorbidities that may trigger the hypertensive episode and subsequently cerebral edema. In most patients, the systolic blood pressure is raised above 160 mmHg but in some cases it may be on the normal side or slightly elevated [5].

The common comorbidities associated with PRES are acute kidney injury (AKI) and eclampsia [1]. But it has also been found in not-so-common scenarios like Guillain Barre syndrome [6], illicit drug use [7], autoimmune diseases such as SLE and immune thrombocytopenic purpura, and with immunosuppressive drugs such as cyclosporine and tacrolimus [8].

If we talk about the neurological involvement in SLE, there is a wide range of manifestations that can be seen in those patients [9]. These range from idiopathic intracranial hypertension to seizures which can be very common as 14-25% of SLE patients are found to have seizures [10]. Another common neurological manifestation in lupus patients is stroke or transient ischemic attack (TIA), which is seen in about 5-20% of SLE patients [11]. Apart from this, psychiatric and cognitive disturbances and aseptic meningitis can also be seen in lupus patients [12].

Although less common, PRES can also be the initial manifestation of neurolupus [13]. In a retrospective study by Gatla et al., five SLE patients were diagnosed with PRES. Of these five patients, the most common presentations were headache, seizures and confusion. The imaging revealed intracerebral hematoma in one of the patients while two of the patients had diffuse petechial cerebral hemorrhages. All of them recovered without any significant neurological deficit with a mean hospital stay of 11 days [14]. 

## Conclusions

PRES can be one of the manifestations of neurolupus in SLE patients. Alternatively, we can say that SLE should be kept in the differentials in patients with PRES. PRES is not uncommon in SLE and its presentation is no different than PRES from other causes. However there are certain conditions in SLE patients that may mimic the presentation of PRES; these include cerebral thrombotic events due to antiphospholipid antibody syndrome (APLA) or progressive multifocal leukoencephalopathy due to certain immunosuppressive medications. Since PRES has a reversible nature, prompt diagnosis is important and for this we should keep a low threshold for imaging. If imaging reveals findings associated with PRES, aggressive treatment with antihypertensives and osmotic agents should be started to decrease cerebral edema and prevent a permanent neurological deficit.
